# Insulin and bone health in young adults: The mediator role of lean mass

**DOI:** 10.1371/journal.pone.0173874

**Published:** 2017-03-21

**Authors:** Ana Torres-Costoso, Diana P. Pozuelo-Carrascosa, Celia Álvarez-Bueno, Asunción Ferri-Morales, Jose Miota Ibarra, Blanca Notario-Pacheco, Vicente Martínez-Vizcaíno

**Affiliations:** 1 Universidad de Castilla-La Mancha. School of Nursing and Physiotherapy, Toledo, Spain; 2 Universidad de Castilla-La Mancha. Health and Social Research Center, Cuenca, Spain; 3 Universidad Autónoma de Chile. Facultad de Ciencias de la Salud., Talca, Chile; University of California Davis, UNITED STATES

## Abstract

**Background:**

The positive relationship between lean mass (LM) and bone health is well known, but a positive association between insulin and LM has also been described. Insulin has some anabolic properties on bone through the stimulation of osteoblast differentiation, yet the role of LM as a confounder or mediator in this relationship remains uncertain.

**Objective:**

To examine whether the association between insulin levels and bone health is mediated by LM.

**Methods:**

A cross-sectional study was conducted at the Castilla La Mancha University (Spain) involving 466 young adults (113 young men; 19.5±2.3 years). LM and total-body bone mineral content (BMC) were measured by dual energy x-ray absorptiometry, and insulin was measured in fasting serum samples.

**Results:**

Young adults with high total LM had higher values of total-body BMC than their peers after controlling for age and sex, this relationship persisted after adjusting for insulin levels (p<0.001). In mediation analyses, insulin levels were positively associated with total-body BMC (b = 0.05; p<0.001) and total LM acted as an intermediate variable, attenuating the association between insulin levels and total-body BMC (b = -31.98; p>0.05) as indicated by Sobel test values for indirect effect (z = 4.43; p<0.001).

**Conclusions:**

LM plays an important role in the relationship between insulin levels and bone health, in such a way that while increases in LM have a positive influence on bone health, they are also negatively associated with insulin levels.

## Introduction

Osteoporotic fractures are a major cause of morbidity and mortality in developed countries [1]. These fractures are clinical consequences of osteoporosis, a systemic skeletal disease that contributes to bone fragility [2]. Evidence consistently supports that peak bone mass, defined as the amount of bone acquired at the end of skeletal development that usually occurs between the second and third decades of life [3,4], is an important determinant of lifelong skeletal health and a key determinant of future fracture risk during adulthood [5,6].

Bone mass variability is determined by several factors, including genetics, mechanical and endocrine factors [7–9]. Lean mass (LM) is considered the best predictor of bone mineral content (BMC) in adolescents and young adults [10,11], though its relationship with bone health is complex due to the multiple associations in which this body composition component is involved. On the other hand, LM is an excellent indicator of bone mechanical stimulation and its changes are highly correlated with bone health [12–14]. A linear relationship between LM and BMC during growth has been reported, forming bone and muscle a functional unit [15,16]. Moreover, LM is positively associated with body weight and fat mass, and both influence bone turnover [13,17]. Lastly, LM is associated with insulin levels, because insulin can stimulate amino acid transport and protein synthesis [18,19] by inhibiting proteolysis [20] in skeletal muscle. These physiological effects have been used for therapeutic purposes and to increase muscle mass in individuals involved in sport activities [21].

Insulin has an anabolic effect on bone [22,23], through the stimulation of osteoblast differentiation, which enhances production of osteocalcin [24]. Even though the role of LM as a confounder or mediator in the relationship between insulin and bone remains uncertain, it seems plausible that the metabolic effect of insulin resistance on muscle mass could influence bone health, since a positive association between lean mass and bone outcomes is well known.

Although the relationship between insulin and LM has been repeatedly described, no studies have jointly examined the association of these predictors with bone outcomes. Furthermore, most published studies have been conducted using statistical multivariate procedures (ANCOVA, multiple linear regression or logistic regression) in order to control for potential confounders, but these statistical procedures are unable to distinguish between confounding and mediating variables.

The present study aimed to determine whether the relationship between insulin and bone health is mediated by LM in young adults.

## Subjects and methods

### Study design and participants

This was a cross-sectional ancillary study of a previously conducted population-based study [25,26] aimed at assessing changes in lifestyle and cardiovascular risk that occur during an individual’s time at university. A study, which included all first-year university students of the 2009–2010 academic year from the Castilla-La Mancha University in Cuenca Campus, Spain, were performed. A total of 770 students were invited and 683 (88.7%) agreed to participate. In this report, we use data from a subsample of 466 university students in which BMC (by dual energy x-ray absorptiometry [DXA]) was measured. The young adults included in the data analysis for this study did not differ in age, sex or parental socioeconomic status from the whole sample of young adults participating in the trial.

The study protocol was approved by the Clinical Research Ethics Committee of the Hospital Virgen de la Luz in Cuenca, once participants were informed verbally and in writing, they were asked to sign a consent form as a condition to participate in the study. Because there were no participants aged less than 18 years and that is the legal age in Spain, written informed consent was individually obtained from each participant. Documents with the signed consent were recorded. The Ethics Committee approved the study protocol including permissions and informed consent documents.

### Study variables

#### Anthropometry

Weight was measured twice with the subject barefoot and wearing light clothing using a Seca-770 scale. Height was measured twice with the subject barefoot and upright, with the sagittal midline at the midline of the stadiometer, using a Seca-222 wall-mounted stadiometer. Body mass index was calculated as weight in kilograms divided by the square of height in meters (kg/m^2^) using the means of the weight and height measurements.

#### Body composition

The young adults were scanned in the supine position using DXA (Lunar iDXA, GE Medical Systems Lunar, Madison, WI 53718, USA). The analyses were performed using enCoreTM 2008 software version 12.30.008. DXA equipment accuracy was checked daily before each scanning session using the GE Lunar calibration phantom, as recommended by the manufacturer. All scans were performed at high resolution by the same trained researcher. Bone mineral density (BMD) (g/cm2), fat mass (g), and LM (g) were obtained for each individual from total analysis of the whole body scan. BMC (g) and LM were calculated as follows: BMC = [BMD x area] and LM = [total mass—(fat mass + BMC)]. For all analyses, total LM was categorized as follows: low (1^st^ quartile), medium (2^nd^ and 3^rd^ quartiles) and high (4^th^ quartile).

#### Serum biochemistries

Insulin and glucose were measured in serum blood samples and they were collected via a cubital vein puncture under standard conditions [27] between 8:15 and 9:00 AM, after at least 12 hours of fasting. The samples were processed in a COBAS C711 system from Roche Diagnostics, blood glucose concentration was determined by the hexokinase method and blood insulin concentration was determined by the one-step chemiluminescent microparticle immunoassay and processing on a platform composed of two ARCHITECT i2000SR systems from Abbott Laboratories. The variation coefficient of fasting insulin ranged from 2.47 to 3.34%, at the lower and higher levels, respectively. For all analyses, insulin levels were categorized as follows: low (1^st^ quartile), medium (2^nd^ and 3^rd^ quartiles) and high (4^th^ quartile).

### Statistical analysis

Both statistical (Kolmogorov–Smirnov test) and graphical methods (normal probability plots) were used to examine fitting to a normal distribution for each continuous variable. Insulin levels were not normally distributed and were log transformed.

ANCOVA models were used to test mean differences in total-body BMC by insulin level categories. Age and sex were covariates in model 1, and age, sex and total LM were covariates in model 2. Similarly, when we used total LM categories as fixed factors, we used as covariates age and sex in model 1, and age, sex and insulin levels in model 2.

**Fig 1:**
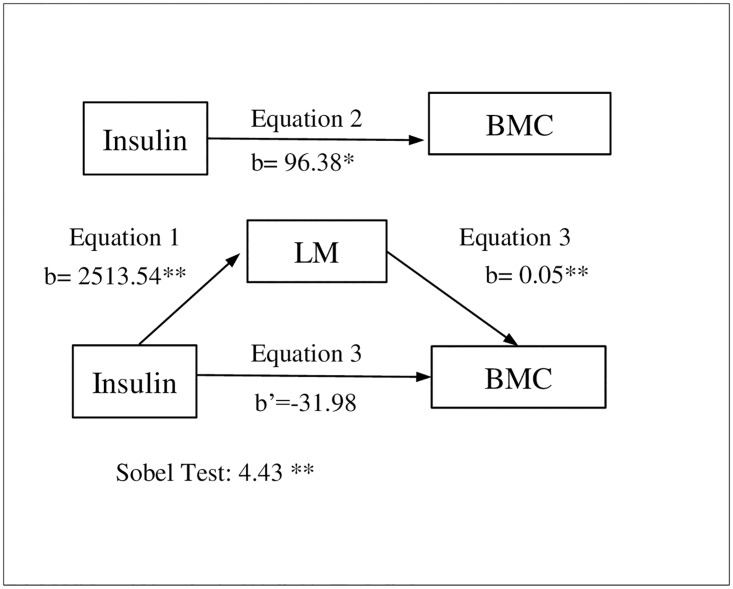
Simple mediation models of the relationship between insulin and total-body Bone Mineral Content (BMC) using total Lean Mass (LM) as a mediator, and controlling for age and sex. *p<0.050; **p<0.001.

Linear regression analyses were conducted to test the potential mediating effect of total LM in the association between insulin levels and total-body BMC, following the criteria outlined by Baron and Kenny [28] namely: 1) the independent variable must be significantly related to the mediator, 2) the independent variable must be significantly related to the dependent variable, 3) the mediator must be significantly related to the dependent variable, and 4) the association between the independent and dependent variables must be attenuated when the mediator is included in the regression model. In addition, we tested mediation effect using the steps outlined by Sobel [29]: 1) we estimated the attenuation or indirect effect (i.e. the effect of the independent variable on the mediator from the first regression model multiplied by the effect of the mediator on the dependent variable obtained from the third regression model), and 2) we divided the indirect effect by its standard error and performed a z-test under the null hypothesis that the indirect effect is equal to zero. The regression model was adjusted for age and sex (Fig 1).

Simple mediation models were estimated using the PROCESS macro for SPSS. This macro uses bootstrapping methods as recommended by Preacher and Hayes [30] for testing mediation hypotheses (we used a resample procedure of 10,000 bootstrap samples). In the mediation analysis with the three variables, BMC, insulin and LM were used in their original quantitative scale. The study data are shown in S1 File. Statistical analyses were performed with SPSS-IBM (Software, v.19.0 SPSS Inc., Chicago, IL, USA) and the level of significance was set at α = 0.05.

## Results

Descriptive characteristics (mean ± standard deviation [SD]) of the study sample are shown in Table 1. All variables differed significantly by sex except insulin levels, fasting glucose and age. Table 2 shows the mean-adjusted differences in total-body BMC by insulin levels and total LM categories, after controlling for potential confounders. Young adults with high insulin levels showed higher total-body BMC than their peers, though the differences did not reach statistical significance after controlling for age and sex (model 1), and additionally for total LM (model 2). Moreover, young adults with high total LM had significantly higher total-body BMC than those with low total LM after controlling for age and sex (model 1), and also when insulin levels were controlled for (model 2).

**Table 1:** Descriptive characteristics of the study sample (mean ± SD).

	All (466)	Boys (113)	Girls (353)	p
Age (years)	19.5±2.3	19.8±2.3	19.4±2.2	0.094
Body mass (kg)	62.1±12.1	73.3±11.8	58.6±9.8	**<0.001**
Height (cm)	166.0±8.4	175.9±7.5	162.8±5.6	**<0.001**
BMI (Kg/m^2^)	22.5± 3.5	23.6±3.3	22.1±3.5	**0.001**
Total LM (g)	41516.7±8898.5	54518.8±6742.3	37354.5±4300.5	**<0.001**
Insulin levels (IU/mL	8.2±3.5	7.7±2.9	8.3±3.7	0.100
Fasting glucose (mmol/l)	86.7±15.4	88.4±0.6	86.1±0.9	0.189
Total body BMC (g)	2446.6±453.5	3010.2±0.5	2266.2±267.2	**<0.001**

*BMI* body mass index; *LM* lean mass; *BMC* bone mineral content.

**Table 2:** ANCOVA models comparing means of total-body Bone Mineral Content (BMC) by insulin levels and total Lean Mass (LM) categories in young adults.

BMC (g)								
	Insulin levels				Total lean mass			
	Low	Medium	High	p	Low	Medium	High	p
	n = 113	n = 224	n = 109		n = 116	n = 234	n = 116	
Model 1	2590.94±35.17	2636.88±25.23	2697.05±41.90	0.158	2046.82±25.15	2421.63±40.14	2912.25±37.45 ^a^^,^ ^b^	**<0.001**
Model 2	2417.16±22.65	2405.54±17.81	2394.47±28.22	0.801	2042.22±25.95	2426.10±40.30	2912.08±38.24 ^a^^,^ ^b^	**<0.001**

Covariates for insulin levels: Model 1 (age and sex); Model 2 (Model 1+ total lean mass).

Covariates for total lean mass: Model 1 (age and sex); Model 2 (Model 1+ insulin levels).

Superscript letters indicate statistical significance (p≤0.050) for post-hoc hypothesis test determinates by using the Bonferroni correction for multiple comparisons:

^a^ High>Medium>Low;

^b^ High>Low

### Simple mediation analysis

We tested the mediator role of total LM (Fig 1) in the relationship between insulin levels and total-body BMC. The relationship between insulin levels and total LM was positive (b = 2513.54; p<0.001) in the first regression equation, and between insulin levels and total-body BMC (b = 96.38; p<0.050) in the second regression equation. In the third regression equation, the relationship between total LM and total-body BMC was positive (b = 0.05; p<0.001), though between insulin levels and total-body BMC it was attenuated when the mediator was included in the regression model (b = -31.98; p>0.050). Thus, total LM acted as a mediator of the relationship between insulin levels and total-body BMC, as shown by the Sobel test for indirect effect (z = 4.43; p<0.001). The percentage of total effect mediated by total LM was 26.8%.

## Discussion

To the best of our knowledge, this is the first study in young adults analysing whether total-body BMC levels are related to insulin levels regardless of total LM or, conversely, whether the latter acts as mediator in the association between insulin levels and total-body BMC. The main findings of this study are: (1) young adults with high total LM have more total-body BMC than those with lower total LM after controlling for relevant confounders, including insulin levels; and (2) total LM is a total mediator in the relationship between insulin levels and total-body BMC.

Insulin-like growth factor and insulin play an important role in muscle development [31]. The anabolic actions of insulin are of interest to people, such as athletes and body builders who want to increase their muscle mass, and to those concerned with preventing sarcopenia. It is well known that insulin inhibits protein catabolism, and increases the synthesis of glycogen and proteins in muscle, promoting the entry of glycogen and amino acids into muscle cells [32,33]. In addition, physiological hyperinsulinemia has been shown to enhance the activity of amino acid transport and protein synthesis in muscle mass [18]. Accordingly, our study shows a positive relationship between insulin and lean mass.

The mechanostat theory describes muscle as a mediator that transfers the ground reaction forces and the forces generated during muscle contractions to bone [34]. Literature consistently considers LM as the best predictor of bone health in adolescents and young adults [10,35]. Similar to previous results, our findings show that total-body BMC levels are positively associated with LM in young adults.

The role of insulin-like growth factor in the regulation, development and homeostasis maintenance of bone is well known [36,37], as well as the fact that its homologue, insulin, has some anabolic properties for bone. Insulin may work by stimulating osteoblast differentiation, which in turn would enhance the production of osteocalcin [24,38]. Moreover, insulin might exert an effect on bone cells through direct binding to the insulin receptor, which has been detected in primary human osteoblasts differentiated from bone marrow-derived mesenchymal stem cells [39]. Our data in is line with previous reports that show high insulin levels were related with higher total-body BMC, though the differences did not reach statistical significance after controlling for confounders such as LM, which might explain this relationship.

There is consistent evidence regarding the bivariate association of LM with both insulin [8,24,38,40] and bone [11,41,42]. Likewise, the relationship between insulin and bone in humans has been established [22,43]. In addition, a recent study has showed that lean body mass is an important intermediary factor in the insulin-like growth factor 1 and bone relationship in premenarcheal girls [44]. However, it has not been fully illustrated whether LM acts as a confounder or as a mediator in the association between insulin levels and total-body BMC in young adults. Our study confirms the independent relationship between LM and insulin levels with total-body BMC, and it clarifies the mediating role of LM in the relationship between insulin levels and total-body BMC.

The current study has several limitations that should be acknowledged. First, the cross-sectional design does not allow us to make cause–effect inferences and no study design has the statistical power or is as free of bias as prospective intervention studies. However, in terms of feasibility, such a study design would require a large sample size or a long follow-up period, apart from the ethical considerations for these kinds of studies. This is probably why most mediation analysis are cross-sectional [45–47]. Second, our results are based on analyses of insulin levels measured through fasting insulin rather than an index of insulin resistance such as the homeostasis model assessment of insulin (HOMA-IR). However, insulin levels are well correlated with HOMA-IR (r = 0.85; p<0.001) [48]; thus, in the current population, HOMA-IR offers no advantages in evaluating insulin resistance. Finally, the relationships analysed were likely influenced by more than one mediator variable; future studies using structural equation procedures might be useful to more specifically clarify the potential mediator role of each factor.

## Conclusions

Our data are relevant from a clinical perspective as they disclose that the association between insulin and bone mass in young adults seems to be mediated by LM. Thus, LM may influence not only glucose metabolism but also bone health.

## Supporting information

S1 File Study data. (XLSX)
